# Structure of Musical Dislikes: Findings From Multiple Genres and Countries

**DOI:** 10.1111/nyas.70408

**Published:** 2026-10-01

**Authors:** Julia Merrill, Klaus Frieler, Amanda E. Krause

**Affiliations:** ^1^ Max Planck Institute for Empirical Aesthetics Frankfurt am Main Germany; ^2^ Institute of Music University of Kassel Kassel Germany; ^3^ Department of Psychology James Cook University Townsville Queensland Australia

**Keywords:** displeasure, emotional arousal, music perception, musical taste, preference

## Abstract

Musical taste shapes social life and personal identity, yet research has focused mainly on why people like music, leaving the equally powerful phenomenon of musical dislikes largely unexamined. Building on recent work identifying core reasons for musical aversion, this study examined the psychological structure, genre associations, individual difference predictors, and cross‐cultural variation of musical dislikes across 49 genres in Australia, China, Singapore, and the United States (*N* = 1078). Using network psychometrics and latent variable modeling, we identified five dimensions underlying aversion: Displeasure, Complexity, Sentimentality, Social Incongruence, and Mainstream orientation. These dimensions mapped onto genre‐level signatures and cross‐cultural listener profiles. Behavioral models showed that only Displeasure and Social Incongruence predicted stronger dislike intensity, indicating that affective discomfort and identity mismatch drive aversion. Complexity and Sentimentality were sensitive to cultural context, whereas basic Displeasure was universal. Individual traits—personality and musical engagement styles—explained more variance in dislike mechanisms than country‐level backgrounds. By mapping the systematic architecture of what listeners reject, this study offers a novel, comprehensive framework for understanding how individuals navigate cultural boundaries, regulate affect, and signal identity through active avoidance, proving that musical dislikes are not merely the absence of liking but a structured and meaningful component of musical taste.

## Graphical abstract

While research historically focuses on what listeners like, this cross‐cultural study examines the structured architecture of musical aversion across 49 genres. Combining network psychometrics and factor modeling, we uncover five interconnected mechanisms of dislike: Displeasure, Complexity, Sentimentality, Social Incongruence, and Mainstream orientation. The findings demonstrate that musical dislikes form a systematic, culture‐modulated framework central to affect regulation and social identity signaling.

## 1 Introduction

Musical taste is a central aspect of human experience, shaping social identity, personal expression, and everyday behavior. Traditionally, research in musical taste has focused predominantly on likes or preferences for certain types of music [1, 2, 3, 4]. Recent studies, however, highlight that musical dislikes are not merely the absence of preference but serve important psychological and social functions, including social distinction, identity construction, and emotion regulation [5, 6, 7]. Therefore, a comprehensive understanding of musical taste must incorporate negative evaluations and the reasons behind them. To comprehensively map this phenomenon across diverse contexts, this paper investigates how the multidimensional mechanisms of musical dislikes are structured, how they vary across genres and intensity levels, and how they are shaped by individual traits and cultural backgrounds.

Understanding how and why listeners develop aversions to specific musical genres requires an integration of multiple intersecting disciplinary lenses. Therefore, the following sections systematically review the psychological dynamics of cognitive and affective processing, the socio‐ideological contexts of cultural distinction, baseline cross‐cultural variations, and stable individual dispositions.

### 1.1 Aesthetic, Cognitive, and Affective Mechanisms

Existing research offers complementary frameworks for understanding why listeners dislike certain types of music, each emphasizing different aspects of how people evaluate and reject musical genres. The reciprocal feedback model proposes that musical behavior—including musical preference—emerges from reciprocal interactions among the listener (personality, identity, age, stable musical preferences, attitudes), the listening situation (social context, cultural setting, purpose, environment), and the music itself (structural features, genre, complexity, familiarity) [8]. From an aesthetic and cognitive perspective, these interactions are fundamentally guided by how individuals process structural density and information load, which can trigger feelings of over‐ or understimulation [9, 10, 11, 12]. Variations in structural elements, particularly tempo, actively modulate these cognitive processing loops by simultaneously driving physiological arousal, structural clarity, and subjective liking [13, 14]. The key idea is that preferences are shaped by ongoing feedback loops between these three components, across both short and long timescales.

In a similar vein, a model on judgments of musical value arises from three interacting dimensions: an objective dimension, referring to the object itself (e.g., the music); a subjective dimension, referring to the person who experiences it; and a social dimension, shaped by the environment and contextual factors [15, 16]. Building on this framework and focusing specifically on musical dislikes, we have previously proposed a more focused typology of musical dislikes that distinguishes between intrinsic reasons, referring to judgments based on the sonic or structural properties of the music, and extrinsic reasons, arising from the meaning listeners attribute to music [6]. Extrinsic reasons include social reasons, such as ideological mismatches or negative associations with fan groups, and emotional extrinsic reasons, in which the emotional content or expressivity of music is experienced as inappropriate, overwhelming, or undesired.

Psychological and neuroaesthetic frameworks of musical evaluation suggest that these distinct aversive reactions are structurally divided into immediate affective or sensory responses and higher‐order evaluative appraisals [17, 18, 19]. Basic sensory affect operates as a vital baseline that actively grounds and anchors subsequent higher‐order aesthetic evaluations [19, 20]. Furthermore, integrative psychological models of music evaluation emphasize that identity‐based evaluations and emotional intensity assessments represent distinct psychological pathways [21, 22]. These frameworks highlight that musical dislike is often not simply about sound qualities but about how these are interpreted in relation to the self and one's social world.

The present study builds on these frameworks by interpreting reasons for musical dislikes as reflecting distinct aesthetic, cognitive, and affective psychological mechanisms that contribute to aversion. Importantly, these frameworks are highly compatible: intrinsic (sound‐based) aspects map directly onto (more or less) objective musical properties, whereas extrinsic (meaning‐based) aspects encompass subjective and social dimensions. The psychological mechanism approach bridges these models by explaining how the listener integrates object‐related and social dimensions through biological and enculturation processes. Ultimately, these mechanisms serve emotional and social regulative goals—such as preventing sensory or cognitive displeasure on the one hand, and avoiding undesired social associations on the other.

### 1.2 Socio‐Ideological Contexts and Mainstream Judgments

Musical taste has also been conceptualized in sociological terms. Bourdieu's theory of social distinction [23] and subsequent studies on omnivorousness suggest that preferences and dislikes function as markers of social and cultural identity, allowing groups to differentiate themselves from others [24, 25, 26, 27]. All musical genres are culturally coded, but some more strongly than others—rap and gospel with youth and Black communities, metal with White working and lower‐middle‐class audiences, and techno with urban youth [28]—creating stereotypical associations that may influence both likes and dislikes [29, 30, 31, 32]. Within these social practices, aversion can manifest either as a deep, affectively potent rejection of a genre that threatens one's social identity and group boundaries [33] or as a more detached political and ideological attitude, such as rejecting commercialized music from the mainstream [23, 34].

These identity‐based boundary practices depend heavily on specific subcultural markers and localized genre hierarchies. For instance, the rejection of elite genres like opera is often driven by perceived social mismatch with highbrow culture [35], whereas the rejection of extreme genres like heavy metal is frequently tied to intense subcultural aesthetics (e.g., intense imagery, loud sonic styles, and specific group behaviors) and defensive moralized discourses [36, 37, 38]. Conversely, aversion to highly commercialized or culturally saturated genres like pop or K‐pop is often triggered by perceived structural banality, overexposure, and media saturation [34, 39, 40, 41].

A central question is whether these reasons operate as isolated factors or form a coordinated system of interrelated mechanisms, as proposed by the reciprocal feedback model. Network‐theoretic approaches have proven useful in modeling psychological constructs as interconnected processes rather than single latent traits [42]. Applied to music, such approaches can illuminate how displeasure, complexity, emotionality, social meaning, and other aversive responses and reasons relate to one another—whether they overlap, reinforce each other, or remain largely independent [6].

### 1.3 Cross‐Cultural Variations and Baseline Universals

While prior research has explored qualitative reasons for musical dislikes—including simplicity, repetitiveness, perceived inauthenticity, emotional intensity, or social incongruence—these studies have been limited in sample size or scope and rarely examine the interaction between reasons, musical genre, and intensity of dislike [7, 43, 44, 45, 46]. Another limitation is the strong Western and WEIRD (Western, educated, industrialized, rich, democratic) bias in existing work. Research on music perception shows a mixture of universals and cultural variation. When it comes to universalities of music perception, listeners across cultures, for example, respond similarly to acoustic and musical cues such as roughness or tempo [47, 48], and cross‐cultural corpus analyses reveal shared structural features in musical systems worldwide [49]. This suggests that basic sensory displeasure may function as a universal human baseline that operates relatively independently of specific cultural backgrounds [50].

At the same time, cultural factors—such as learned rules for emotional expression and socioeconomic status—heavily influence or even shape subjective taste [51]. Cultural norms deeply shape preferences for emotional intensity [52], tolerance thresholds for emotional expressivity [53], and expectations for structural density, tonality, and temporal regularity [54, 55, 56, 57]. Furthermore, macro‐level cultural contexts systematically shape the social meaning of genres [58], altering expectations for emotional display rules [59]. In individualistic societies, musical preferences are leveraged for identity and personality signaling, whereas in collectivistic societies, the demand for unique, individualistic taste distinction is significantly reduced [60, 61, 62]. Despite this, virtually no studies have examined whether reasons for disliking music differ across cultures. It remains unknown which aversive mechanisms are culturally widespread and which reflect culture‐specific norms, ideologies, and exposure patterns.

### 1.4 Individual Differences and Dispositional Traits

Individual traits play a central role in shaping how people perceive and evaluate music more generally, and these same traits are likely to influence musical dislikes. Research on personality and musical preference consistently shows that traits such as openness to experience (weakly) predict liking for complex, intense, or unconventional music, whereas extraversion and agreeableness relate to preferences for rhythmic, energetic, or emotionally positive genres [63, 64, 65], potentially driven by underlying variations in cortical arousal sensitivity [66].

Musical sophistication—including training, perceptual abilities, and engagement—has been linked to greater tolerance for complexity, structural irregularity, and unfamiliar musical systems [67], as cognitively oriented listeners prefer structural intricacy and novelty over predictable, formulaic musical layouts [68, 69]. Similarly, broader music engagement styles—whether a listener predominantly utilizes music for narrative storytelling, physical stimulation, social bonding, or symbolic identity tracking [70, 71, 72]—likely influence the reasons underlying musical dislikes. Listeners who track narrative functions and lyrics closely develop an increased sensitivity to symbolic markers and potential ideological mismatches [73].

### 1.5 The Present Study

Building on these theoretical foundations, the main research question of this study is as follows: How are the multidimensional mechanisms of musical dislikes structured across diverse cultural contexts, and how are they driven by genre‐specific characteristics, individual traits, and macro‐level cultural backgrounds?

To address this main question, the present study examines reasons for musical dislikes across a large, culturally diverse sample, serving as a direct cross‐cultural extension of an established multidimensional framework [6] and grounded in the reciprocal feedback model [8]. Specifically, we delineate four concrete objectives:

Latent structure (research question [RQ] 1): To examine how the multidimensional structure of musical dislikes is organized across different cultural contexts.
Genres, intensity, and culture (RQ 2): To investigate how reasons for musical dislikes vary across levels of dislike intensity and specific musical genres, and what cross‐cultural variations emerge at the genre level.
Listener profiles (RQ 3): To identify whether distinct cross‐cultural profiles of listeners exist based on their patterns of musical dislikes.
Individual traits versus cultural background (RQ 4): To determine the extent to which stable individual traits versus macro‐level national backgrounds explain variance in these reasons.

To address these objectives, we applied a multistep analytic procedure derived from prior quantitative research [6]. Specifically, we integrated network psychometrics (exploratory graph analysis [EGA]) with traditional latent variable modeling to delineate the overarching multidimensional latent structure of musical dislikes (RQ 1) and examine how these reasons vary across genres, intensity levels, and cultural backgrounds (RQ 2 and RQ 4). This combined approach was explicitly chosen to align with our theoretical framework; because musical dislikes emerge from several dynamical, interrelated psychological mechanisms (affective, cognitive, socio‐ideological), they cannot be fully captured by traditional factor models alone [6]. Furthermore, these analyses were supplemented by a latent profile analysis (LPA) to identify distinct cross‐cultural subgroups of listeners (RQ 3). Grounded in cross‐cultural research on self‐expression and identity construction [60], this person‐centered analytical reasoning allowed us to test whether specific aversion patterns reflect distinct identity processes across cultures.

Overall, the current study contributes to developing a comprehensive framework for understanding why people dislike music. It broadens the scope of musical taste research beyond preference‐centered perspectives, highlighting the layered psychological mechanisms—driven by affective, cognitive, and identity‐based factors—that give rise to aversive responses and demonstrating that musical dislikes constitute a meaningful, structured component of musical engagement.

## 2 Materials and Methods

### 2.1 Participants

A total of 1078 participants (715 female, 283 male, 38 nonbinary, 23 nondisclosure, 19 self‐described) took part in the online survey. The mean age was 22.74 years (SD = 8.41, median = 20). Participants were recruited from several countries, with the largest subsamples from the United States (*n* = 458), Australia (*n* = 253), Singapore (*n* = 240), and China (*n* = 55), and smaller groups from other countries (*n* = 72). About half were employed (*n* = 482); about a third had a trade certificate, diploma, or apprenticeship (*n* = 391), 171 had a bachelor's degree, and 34 had a postgraduate qualification.

The study was approved by the James Cook University Human Research Ethics Committee (application ID number H8921).

### 2.2 Procedure

A customized test battery was set up, using the psychTestR framework [74], running on the Online Testing Service Platform (DOTS) of the German Society for Music Psychology. The survey was advertised through a university student participation scheme and social media. The survey was administered entirely in English. For the Chinese and Singaporean subsamples, recruitment specifically targeted university students and individuals with high English proficiency, eliminating the need for translation and ensuring semantic consistency across all groups. After completing demographic questions, participants indicated their familiarity with 49 musical genres (Table S1) on a 5‐point scale (1 = *not at all familiar*, 5 = *very familiar*). If they reported at least “a little bit familiar” (2 or higher), they rated their liking intensity on a 9‐point scale (1 = *dislike very much*, 9 = *like very much*). Genres disliked (ratings 1–4) were selected for follow‐up by the survey tool. Up to four disliked genres per participant (ideally one per dislike level 1–4) were probed further.

For each selected genre, participants rated their agreement with a set of 42 predefined reasons for dislike on 5‐point Likert scales (1 = *completely disagree*, 5 = *completely agree*; Table S2). The items were previously developed based on a qualitative interview study [5] and probed on a German sample [6]. An open‐ended question then asked participants to compare their most liked and most disliked genres and explain their preferences in three to five sentences. Finally, participants completed additional measures: the Goldsmiths Musical Sophistication Index (GMS, nine items of active engagement) [67], the Music Engagement Test (MET, 23 items of music listening experiences) [75], and the Ten‐Item Personality Inventory (TIPI) [76].

### 2.3 Analysis

All analyses were performed using R version 4.4.3. For analyses of dislike reasons, only ratings of disliked genres (1–4) were considered, reducing the dataset to *n* = 971 participants.

### 2.4 Latent Structure: Network and Factor Analysis

To examine the underlying structure of participants’ musical dislikes and to identify the core latent dimensions based on these responses, we first estimated the dimensional structure of the item set (reasons for dislikes) using EGA as implemented in the *EGAnet* package [77]. EGA estimates a partial correlation network using the graphical LASSO (glasso) and subsequently clusters the items using the Walktrap community detection algorithm. EGA has been shown to outperform traditional factor retention heuristics in determining the number of factors in psychological data [78].

In comparison, we conducted an exploratory factor analysis using the *psych* package [79] with oblimin rotation because the factors were theoretically expected to correlate. To cross‐validate the dimensionality, the number of factors was initially estimated using a parallel analysis and compared to the EGA dimensions. While the parallel analysis suggested an extended structure of nine factors (and six principal components) [80], manual inspection confirmed that dimensions beyond the five core communities suggested by the EGA lacked stable item coverage and theoretical substance. Consequently, an initial minor sixth residual factor (“No Expression”) was evaluated but subsequently discarded from subsequent modeling, leaving five robust dimensions. Factor scores for the final five dimensions were extracted using ten Berge scoring [81], providing scale‐level estimates for each participant based on the full continuous spectrum of all item loadings without applying arbitrary exclusion thresholds. These factor scores were used in subsequent analyses of latent profiles and individual differences.

We also computed bridge centrality indices using the function *bridge()* from the *networktools* package [82]. Bridge centrality identifies items that connect different communities, meaning they may function as cross‐domain indicators linking otherwise distinct facets of musical dislike.

Next, we estimated a partial correlation network of factors using the factor scores as nodes. A Gaussian graphical model (GGM) was computed with *EBICglasso()* from the *qgraph* package [83]. This model captures the unique associations among dislike dimensions while controlling for all other dimensions in the network.

To assess how strongly each dislike reason was embedded within the overall system, nodewise predictability was computed. For each variable, a linear regression model was estimated in which the target node was predicted from all remaining nodes in the network. The resulting *R* ^2^ value reflects the proportion of variance in that node that can be explained by the other dislike reasons.

### 2.5 Genres, Intensity, and Culture: Mixed‐Effects Modeling

To evaluate the behavioral and contextual significance of the identified dimensions, linear mixed‐effects models (LMMs) were fitted across a series of steps. First, to examine the primary drivers of dislike intensity, factor scores were entered as predictors of overall disliking ratings across all genres and participants (*n* = 971), including a random intercept for genre.

Second, to test for genre‐specific signatures and interactions with dislike intensity, separate models were calculated for the 11 most frequently mentioned genres, testing the interaction between dislike intensity and genre for each reason, with participant modeled as a random intercept to account for repeated measures. Third, to identify which specific genres were most strongly associated with each dislike reason across the full spectrum, LMMs were fitted across all 49 genres with fixed effects of genre and dislike intensity, alongside participant and country entering as random intercepts.

Finally, to evaluate cross‐cultural variations at the genre level, models were restricted to the United States, Australia, Singapore, and China across all genres except four that lacked cross‐cultural evaluation data (Dance, House, R&B, Xinyao). These LMMs tested the fixed effects of genre, country, and their interaction (genre × country), while controlling for dislike intensity and including participant as a random intercept.

For the LMMs, Type III Wald *F*‐tests (via the *anova()* function) were conducted to formally evaluate the significance of fixed effects while accounting for the hierarchical structure of the data. Estimated marginal means (EMMs) were computed using the *emmeans* package [84], with pairwise comparisons and confidence intervals extracted for visualization. Tukey correction for *p*‐values was applied for pairwise comparisons. FDR correction was applied to the main effects of interest. The *p*‐values less than 0.05 were considered significant.

### 2.6 Listener Profiles: LPA

To identify distinct subgroups of individuals characterized by shared disliked genres rather than isolated genre evaluations, an LPA was performed on participants’ factor scores derived from the reasons for musical dislikes. Each participant's responses across multiple musical genres were aggregated by computing the mean factor scores per participant, resulting in a single observation per factor per individual. LPA was conducted using the mclust package [87], fitting models with one to six latent classes. The optimal solution was determined based on the Bayesian information criterion, class interpretability, and relative class sizes.

To evaluate whether these listener archetypes were distributed differently across national contexts, the associations between the selected four countries and latent class membership were investigated using Chi‐squared tests with simulated *p*‐values (10,000 replicates) to account for small cell sizes, ensuring robust cross‐cultural inference.

### 2.7 Individual Traits Versus Cultural Background: LMMs and Variance Partitioning

To determine the relative impact of stable personal dispositions versus macro‐level cultural dispositions, LMMs were fitted to examine how reasons for musical dislikes varied as a function of music engagement factors (GMS active engagement; MET physical, affective, social, cognitive, and narrative responses) and personality traits (Big Five: extraversion, agreeableness, conscientiousness, emotional stability, openness to experiences). Dislike intensity, genre, and country were entered as covariates, with participant modeled as a random intercept across all countries.

To formally quantify how much variance in the reasons for musical dislikes is explained by cultural background versus individual traits, we conducted a block‐wise comparison of marginal *R* ^2^ values. To estimate the unique contribution of each predictor block, we created two reduced models: a country‐only model (i.e., excluding all trait variables) and a traits‐only model (i.e., excluding country). Marginal *R* ^2^ values (variance explained by fixed effects) were computed for the full and reduced models using *r.squaredGLMM()* [85]. Block‐specific contributions were then derived as Δ*R* ^2^ for country = *R* ^2^_full_ − *R* ^2^_traits_, and Δ*R* ^2^ for traits = *R* ^2^_full_ − *R* ^2^_country_.

## 3 Results

### 3.1 Latent Structure of Musical Dislikes

EGA and exploratory factor analysis converged on a robust five‐factor structure of musical dislikes: Complexity, Social Incongruence, Displeasure, Mainstream, and Sentimentality (Figure 1).

**FIGURE 1:**
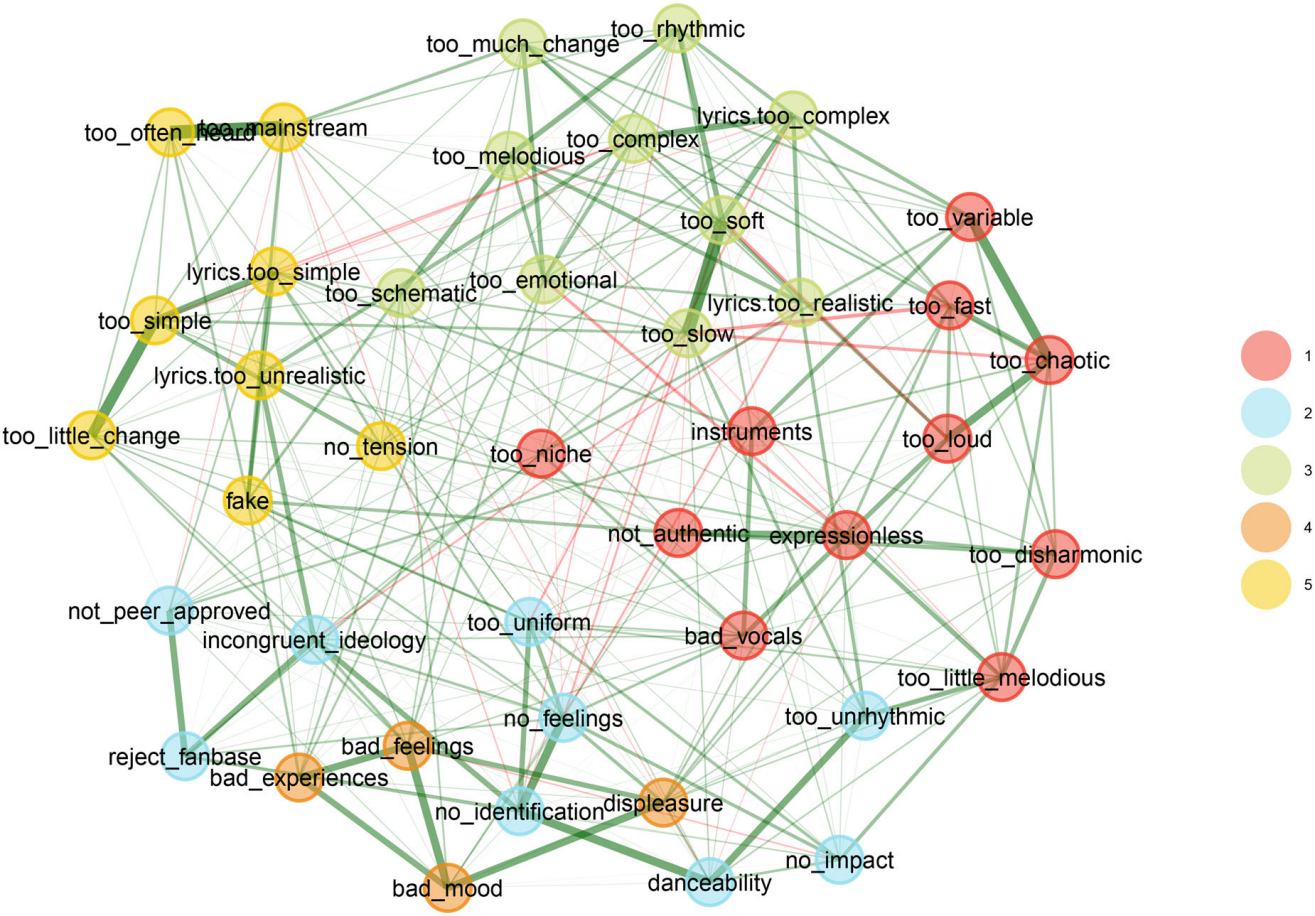
Exploratory graph analysis (EGA) of musical dislike items. Nodes represent individual items describing reasons for disliking music, and edges indicate partial correlations between items. Node colors correspond to the communities detected by the Walktrap algorithm, reflecting clusters of related dislike reasons.

The primary divergence between the EGA and the factor analysis solutions concerns the specific placement of core complexity items (music.too_complex, lyrics.too_complex, and music.too_much_change). In the factor analysis, these items clustered intuitively under a single factor capturing overall feature overload (Complexity), whereas another distinct dimension represented Sentimentality, capturing qualities such as “whiny” or overly “soft” emotional expression (Table 1). This factor‐analytic structure aligns closely with established typologies [6].

**TABLE 1:** Results from the factor analysis of reasons for disliked music.

Item	Complexity	Social Incongruence	Displeasure	Mainstream	Sentimentality	No Expression
*Variance explained*	0.22	0.2	0.18	0.17	0.16	0.07
music.too_chaotic	0.70					
music.too_fast	0.64					
music.too_variable	0.62					
music.too_complex	0.60					
music.too_loud	0.59					
lyrics.too_complex	0.51				0.44	
music.too_much_change	0.39					
music.disliked_instruments	0.39					
music.too_disharmonic	0.38					0.33
social.no_identification		0.69				
emo.no_feelings		0.62				
social.incongruent_ideology		0.61				
social.reject_fanbase		0.56				
body.missing_danceability		0.48				
emo.no_impact		0.44				
music.too_uniform		0.44				
music.bad_vocals		0.38				
social.not_peer_approved		0.35				
music.too_niche		0.27				
emo.bad_mood			0.81			
emo.bad_feelings			0.74			
social.bad_experiences			0.66			
body.displeasure			0.62			
music.too_mainstream				0.66		
social.too_often_heard				0.59		
music.too_simple				0.58		
lyrics.too_simple				0.47		
music.too_little_change				0.47		
social.not_authentic				0.45		
emo.fake				0.39		
lyrics.too_unrealistic				0.38		
music.too_little_tension				0.36		
music.too_soft						
music.too_slow					0.76	
music.too_melodious					0.72	
lyrics.too_realistic					0.48	
music.too_rhythmic	0.37				0.41	
emo.too_emotional					0.39	
music.too_schematic					0.37	
music.too_unrhythmic					0.35	0.34
emo.expressionless				0.38		0.46
music.too_little_melodious	0.34					0.42

*Note*: An initial minor sixth factor (“No Expression”) was excluded from further analysis due to low loadings and insufficient item coverage. Factor loadings <0.30 are suppressed for visual clarity. The underlying latent factors incorporate all items and their full loadings.

Bridge centrality analyses identified key cross‐domain connectors linking these distinct dimensions (Figure 2). The strongest bridge node was too slow (bridge strength = 0.75), demonstrating that tempo‐related aversion serves as a central link between emotional, aesthetic, and complexity‐based dislike mechanisms. Other substantial bridge items included displeasure (0.67), no feelings (0.65), incongruent ideology (0.64), too unrhythmic (0.64), too complex (0.63), emo fake (0.63), and too uniform (0.62), implying that bodily discomfort, lack of emotional engagement, and ideological mismatch function as integrative triggers across multiple domains (all items in Supporting Information).

**FIGURE 2:**
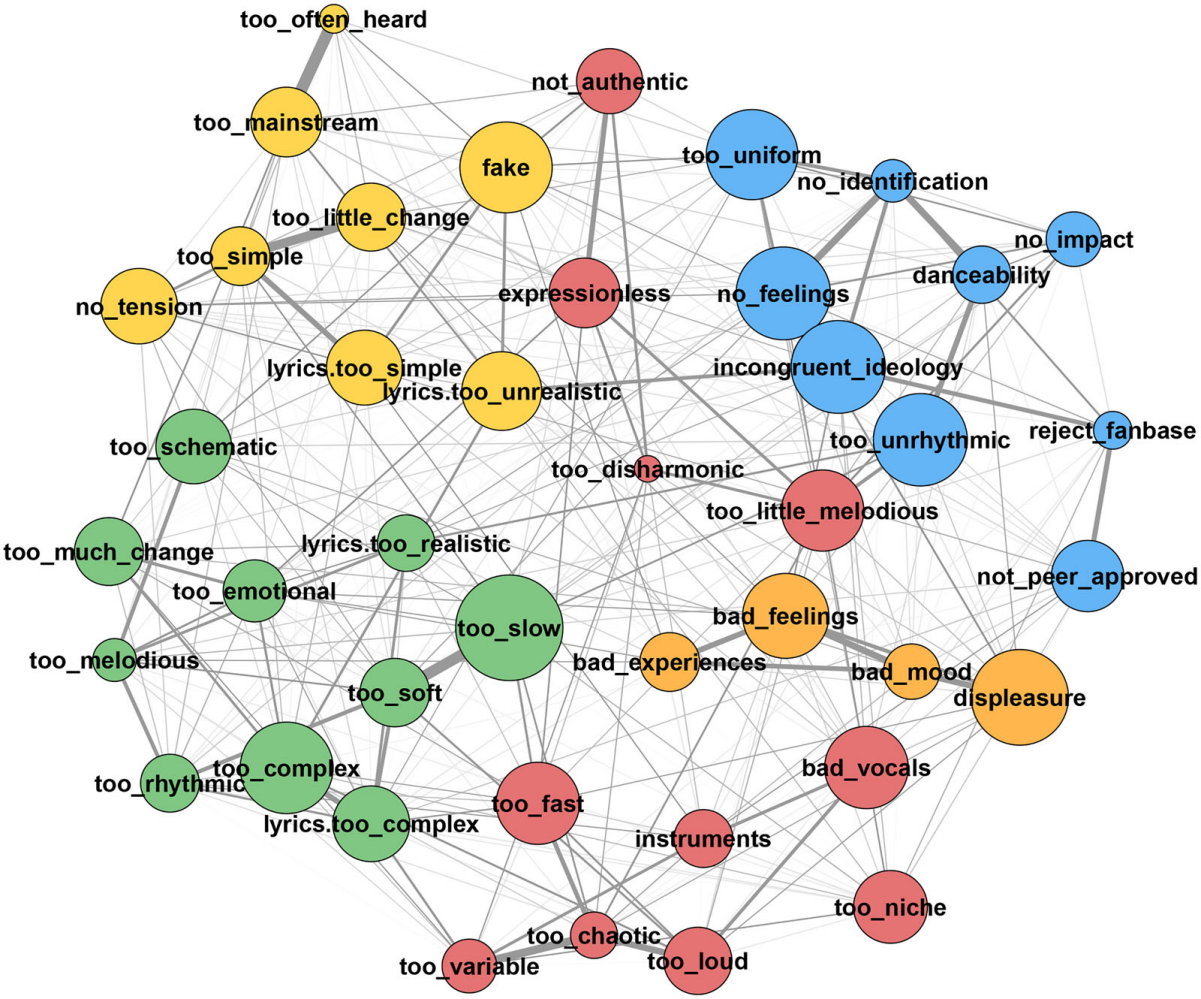
Bridge centrality of musical dislike items. Node size reflects the bridge strength, that is, how strongly a given item connects multiple communities in the EGA network. Larger nodes indicate items that serve as “cross‐community” indicators, linking different dimensions of musical dislike. Node colors correspond to the communities detected by the Walktrap algorithm, reflecting clusters of related dislike reasons.

A partial correlation network estimated the unique associations among the five dimensions while controlling for shared variance (Figure 3). The strongest unique relationship emerged between Complexity and Displeasure, indicating that complexity‐related aversion is intrinsically tied to affective discomfort. Social Incongruence showed a moderate link with Displeasure, whereas its relationships with Mainstream, Complexity, and Sentimentality approached zero, confirming independent socio‐ideological pathways. Additionally, Complexity and Mainstream as well as Displeasure and Sentimentality were negatively associated, while Sentimentality linked moderately with both Complexity and Mainstream.

**FIGURE 3:**
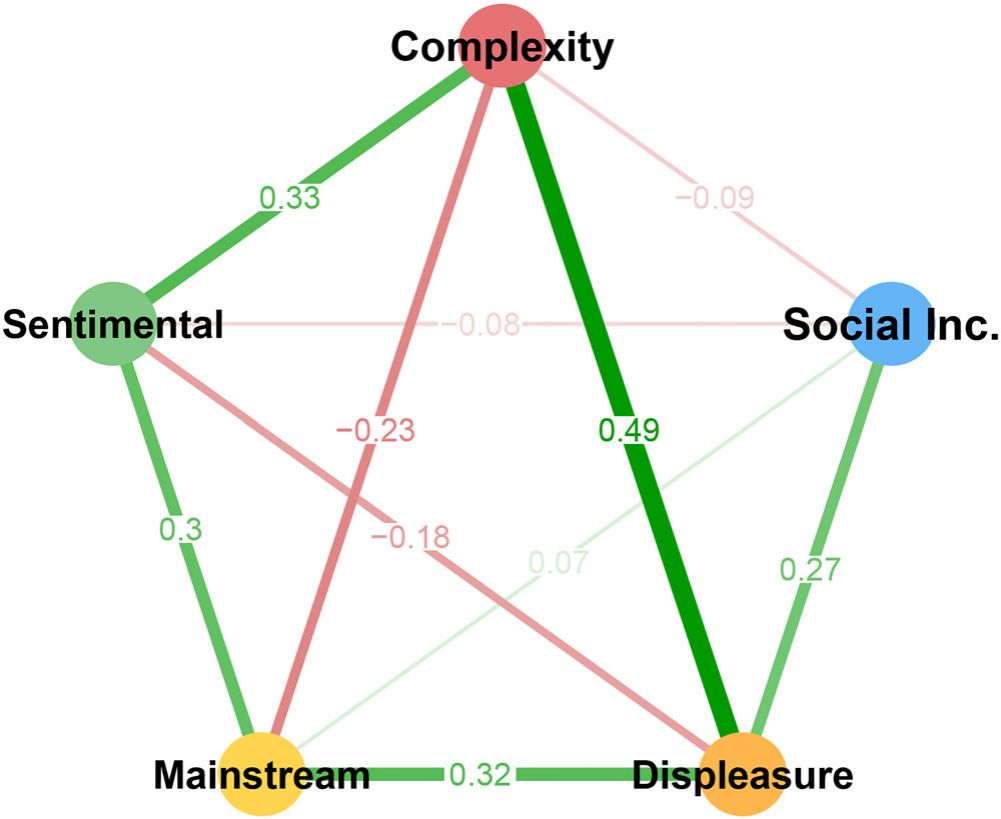
Regularized partial correlation network of factor scores derived from the musical dislike items. Nodes represent latent factors (Complexity, Social Incongruence, Displeasure, Mainstream, Sentimentality), and edges indicate unique associations between factors controlling for all other factors. Edge thickness reflects the magnitude of partial correlations.

Nodewise predictability analyses further supported this architecture. Predictability was highest for Displeasure (*R* ^2^ = 0.41), anchoring its position as the core integrative hub of the network. Complexity also showed moderate predictability (*R* ^2^ = 0.31), while Mainstream (*R* ^2^ = 0.23), Social Incongruence (*R* ^2^ = 0.18), and Sentimentality (*R* ^2^ = 0.17) exhibited lower values. Together, these findings reveal a structured network where Displeasure functions as the central affective hub, while identity‐based and emotional‐mismatch dimensions operate via relatively independent psychological pathways.

### 3.2 Genre Signatures, Dislike Intensity, and Cross‐Cultural Variations

Regarding dislike intensity, the LMM (*R* ^2^_m_ = 0.13) revealed systematic differences in how reasons relate to intensity. Displeasure (*β* = –0.34, *p* < 0.001) and Social Incongruence (*β* = –0.13, *p* < 0.001) were associated with stronger dislike, whereas Sentimentality predicted weaker dislike (*β* = 0.09, *p* < 0.001). Complexity and Mainstream orientation were not significantly related to dislike intensity (*p*s > 0.10).

The LMMs revealed significant main effects of genre for all five factors (Table 2; Figure 4). Significant genre × liking interactions emerged for Complexity and Social Incongruence. Comprehensive analyses across all 49 genres confirmed highly systematic, genre‐specific signatures (all *p*s < 0.001) (Figure S1).

**TABLE 2:** Type III Wald *F*‐tests for the fixed effects of genre, dislike intensity, and country from the linear mixed‐effects models (LMMs).

Contrasts	Complexity	Displeasure	Social Incongruence	Mainstream	Sentimentality
Genres and dislike intensity					
*R* ^2^_m_, *R* ^2^_c_	0.31, 0.52	0.19, 0.41	0.14, 0.45	0.24, 0.46	0.15, 0.5
Genre (*F*, *p* adj.)	15.5, <0.001	2.3, 0.017	2.70, 0.004	6.65, <0.001	7.14, <0.001
Liking (*F*, *p* adj.)	18.8, <0.001	202.7, <0.001	106.63, <0.001	34.77, <0.001	0.01, 0.935
Genre × liking (*F*, *p* adj.)	3.15, <0.001	0.87, 0.559	1.86, 0.047	1.19, 0.294	1.01, 0.652
Genres and Countries					
*R* ^2^_m_, *R* ^2^_c_	0.27, 0.52	0.21, 0.42	0.2, 0.45	0.23, 0.46	0.22, 0.5
Country (*F*, *p* adj.)	8.43, <0.001	1.58, 0.257	3.97, 0.010	2.35, 0.071	5.09, 0.003
Genre × country (*F*, *p* adj.)	1.24, 0.036	0.88, 0.834	1.17, 0.095	1.28, 0.027	1.20, 0.093
AU—CN (*t*, *p* adj.)	−3.91, <0.001		2.63, 0.043		−2.80, 0.026
CN—SG (*t*, *p* adj.)	3.83, <0.001		−3.36, 0.005		2.96, 0.017
CN—US (*t*, *p* adj.)	5.02, <0.001		−2.28, 0.103		3.87, <0.001

*Note*: AU = Australia; CN = China; US = United States; SG = Singapore. *R* ^2^ indicated as marginal (*R* ^2^_m_) and conditional (*R* ^2^_c_) values.

**FIGURE 4:**
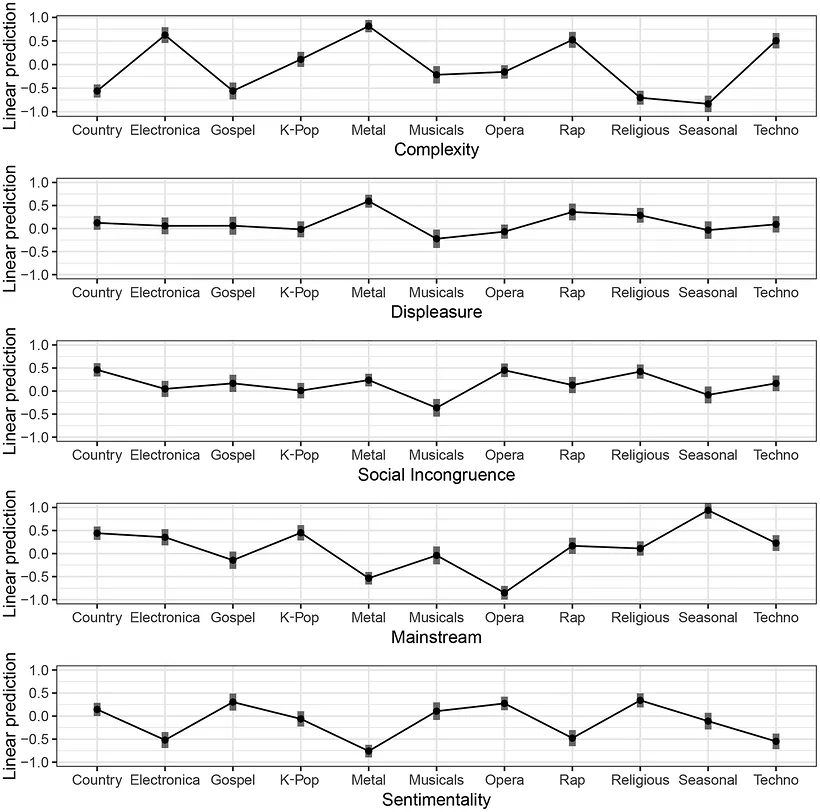
Estimated marginal means of all reasons by genre from linear mixed‐effects models. Values are shown on the response (factor score) scale; error bars indicate 95% confidence intervals.

Perceived Complexity peaked for feature‐dense styles like heavy metal, rap, and electronic music (techno, electronica), but remained irrelevant for “light” genres (gospel, seasonal). Displeasure dominated evaluations of harsh or hard genres (metal, rock, hip‐hop). Social Incongruence was highly selective, applying strongly to religious music, country, and opera. Mainstream orientation was unique to pop‐related contexts (K‐pop, seasonal), while Sentimentality applies to vocal‐heavy, narrative, or slow genres (musicals, opera, gospel, easy listening) but rarely electronic music or hip‐hop.

### 3.3 Cross‐Cultural Variations at the Genre Level

The LMMs examining cross‐cultural variations revealed that broad geographic background interacted distinctively with specific reasons for musical dislikes (Figure 5).

**FIGURE 5:**
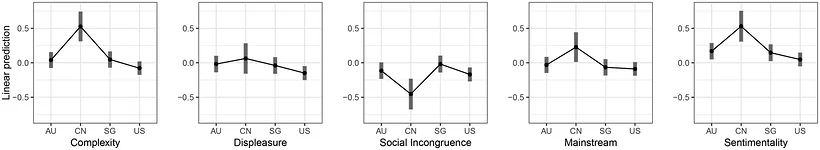
Estimated marginal means of all reasons by country from linear mixed‐effects models. Values are shown on the response (factor score) scale; error bars indicate 95% confidence intervals. AU = Australia; CN = China; US = United States; SG = Singapore.

For Complexity, both the main effect and the genre × country interaction were significant. Post hoc comparisons revealed that Chinese participants perceived disliked music—particularly K‐pop, blues, and soul—as significantly more complex than participants from Australia, Singapore, and the United States (pairwise comparisons can be found in Supporting Information).

For Displeasure, no cultural difference emerged, indicating a potentially universal affective baseline for harsh acoustic stimuli.

For Social Incongruence, Chinese participants reported significantly lower ratings than Australian and Singaporean participants, particularly for opera, jazz, and indie. Conversely, Singaporean participants disliked Getai (a traditional, working‐class Chinese stage performance) significantly more due to social mismatch. A significant genre × country interaction revealed that Chinese participants generally disliked music less than US participants for Mainstream reasons, though they selectively rejected blues and Thai EDM for this reason.

Chinese participants reported significantly higher aversion to Sentimentality than all other national groups, particularly toward country and musicals. These patterns suggest that musical dislikes in China are driven less by social identity concerns and more by internal, aesthetic, and emotional criteria.

### 3.4 Cross‐Cultural Listener Profiles

The LPA identified three distinct classes of listeners based on their musical dislikes (Figure 6, left), with exceptionally high classification certainty (all average posterior probabilities ≥0.99). Socially Driven Dislikers (*n* = 421) were characterized by high sensitivity to Social Incongruence. Balanced Dislikers (*n* = 411) displayed moderate levels across all factors. Emotional Dislikers (*n* = 139) proved particularly sensitive to Sentimentality.

**FIGURE 6:**
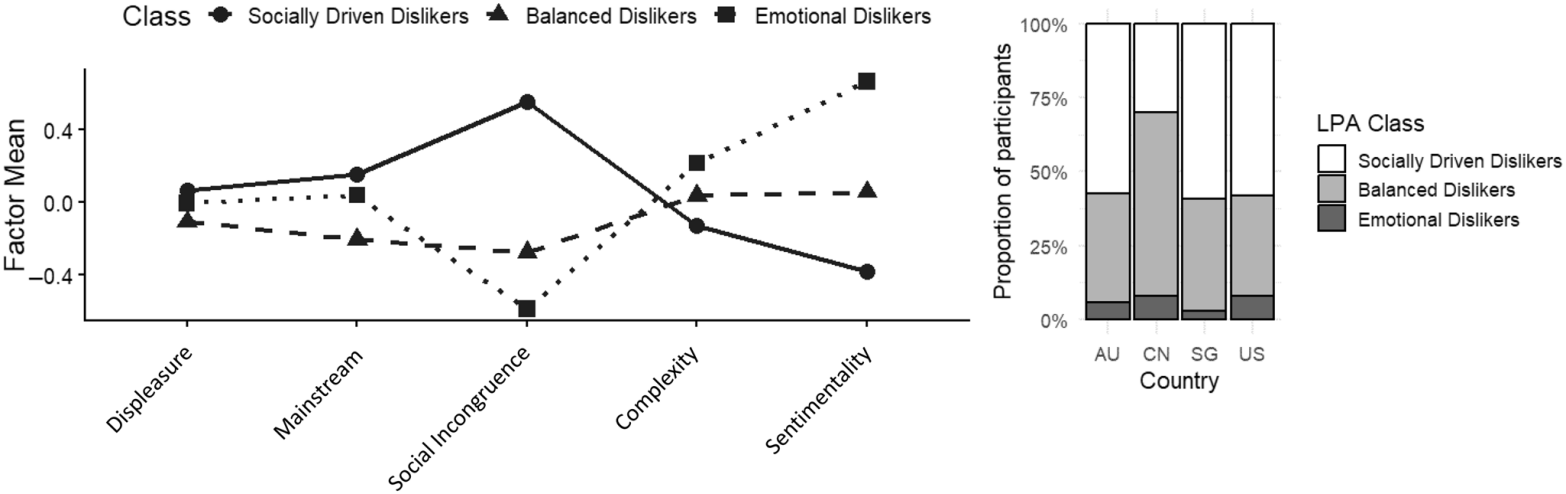
Latent profile analysis. Left: Class‐specific means. Right: Proportion of participants in each latent class per country. AU = Australia; CN = China; US = United States; SG = Singapore.

Cross‐cultural differences revealed a significant association between latent class distribution and country of residence (*χ* ^2^ = 21.88, *p* = 0.002; Figure 6, right). Notably, Socially Driven Dislikers were underrepresented within the Chinese sample.

### 3.5 Individual Traits Versus Cultural Background

LMMs revealed that individual traits—encompassing personality and specific music engagement styles—significantly predicted reasons for musical dislikes (Figure 7). High active engagement (GMS) and high openness to experience acted as cognitive buffers, predicting lower aversion to structural Complexity and Sentimentality. Conversely, analytical and cognitive musical engagement (MET) predicted stronger rejection of music perceived as Mainstream or overly Sentimental. Highly sociable individuals (extraversion) reported stronger aversion to Complex or highly Sentimental music. Regarding explicit content, listeners with strong narrative and storytelling engagement with music exhibited heightened sensitivity to Social Incongruence, reacting strongly to ideological mismatches.

**FIGURE 7:**
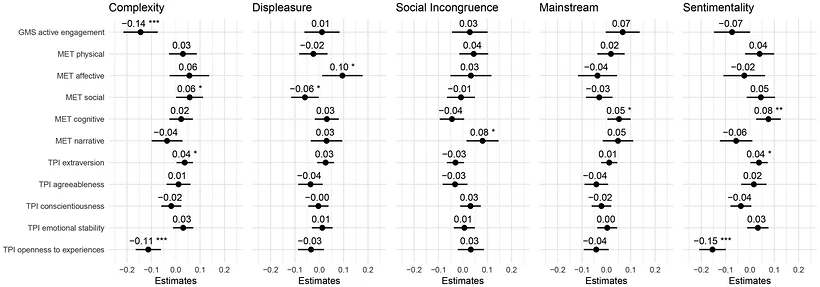
Standardized fixed‐effect estimates from a linear mixed‐effects model predicting reasons (factor scores) with trait variables. Points represent model estimates; lines indicate 95% confidence intervals. GMS = Goldsmiths Musical Sophistication Index; MET = Musical Engagement Test; TPI = Ten‐Item Personality Inventory.

Block‐wise model comparisons quantified the relative impact of macro‐level culture versus individual dispositions. Cultural background (country) explained minimal unique variance (Δ*R* ^2^ = 0.011), whereas individual traits accounted for a larger unique proportion (Δ*R* ^2^ = 0.033). These findings suggest that stable individual psychological profiles and behavioral engagement styles exert a relatively stronger influence on the mechanisms underlying musical dislikes than broad national cultural frameworks.

## 4 Discussion

This study examined the complex landscape of musical dislikes across 49 genres in a culturally diverse sample, using factor analysis, network modeling, and mixed‐effects approaches to uncover the underlying mechanisms of aversion. Across methods, the findings converged on five distinct but interconnected aspects—Displeasure, Sentimentality, Complexity, Social Incongruence, and Mainstream orientation—that jointly shape why listeners reject certain music. Rather than reflecting a single evaluative pathway, musical dislikes emerged as a coordinated system of affective, cognitive, and identity‐related responses, offering a comprehensive framework for understanding how negative reactions to music arise.

### 4.1 Latent Structure of Musical Dislikes

Across methods, the study provides convergent evidence that the five dimensions of reasons for musical dislike reflect distinct yet interconnected psychological mechanisms. Network‐psychometric analyses showed that these dimensions form a connected system rather than isolated traits, with each mechanism contributing unique explanatory power. This structure aligns with theoretical accounts proposing that musical responses arise from overlapping affective, cognitive, and social processes [12, 18, 86].

Predictability analyses further clarified these relationships. Displeasure showed high predictability, suggesting its function as a central affective hub anchoring broader evaluative judgments, consistent with research linking basic affect to higher‐order aesthetic appraisal [19, 20]. In contrast, Social Incongruence and Sentimentality were less predictable, indicating greater conceptual independence and supporting prior distinctions between identity‐based evaluations and emotional intensity judgments [7, 22]. This also suggests a causal relationship, where Displeasure can, at least partly, be considered an effect of the other reasons (including those not covered by our items).

Bridge centrality analyses revealed how these mechanisms interact. Items related to emotional discomfort, complexity, and social meaning connected otherwise distinct clusters, explaining why dislike judgments often involve compound reactions. Tempo‐related aversion—especially music perceived as too slow—emerged as a key bridge, consistent with evidence that tempo simultaneously shapes arousal, structure, and liking [13, 14]. Other bridges highlighted how physiological sensation, cognitive load, and social interpretation jointly contribute to aversion, supporting integrative models of musical evaluation [12, 21].

Together, these psychometric indices reveal an interconnected network where core affective displeasure occupied a central position, while socio‐ideological differentiation and sentimentality operate as distinct, relatively independent motivational pathways underlying musical dislikes.

### 4.2 Genre Signatures, Dislike Intensity, and Cross‐Cultural Variations

Only Displeasure and Social Incongruence were related to stronger dislike intensity, reinforcing their role as core drivers of aversion [5, 6, 7]. Less central mechanisms—Sentimentality and Complexity—did not predict intensity, consistent with findings that perceived emotional expressivity and structural complexity often remain purely evaluative judgments rather than triggering strong affective responses [17].

Hence, the mechanistic pathways revealed by network modeling—affective Displeasure, Social Incongruence, Mainstream evaluation, expressed Sentimentality, and cognitive Complexity—are not theoretical abstractions but correspond to meaningful behavioral outcomes. They map systematically onto variations in dislike intensity, distinct genre‐level signatures, and unique cultural configurations. The integration of network structure, predictability, behavioral intensity, and cultural variation provides compelling evidence that musical dislikes arise from a coordinated but differentiated system of psychological processes.

### 4.3 Aesthetic and Cognitive Excess: Perceived Complexity

The Complexity factor represents a cognitive mechanism distinct from the affective and identity‐related pathways. Across analyses, Complexity behaved differently from all other reasons and was not significantly associated with dislike intensity. This indicates that perceiving a genre as complex reflects cognitive processing demands rather than (emotional) aversion. Earlier studies likewise show that complexity judgments track structural unpredictability and information load rather than valence [9, 11, 12, 68].

Genre‐level patterns aligned strongly with acoustic characteristics: electronica, metal, rap, and techno received the highest Complexity ratings, consistent with their dense textures or rhythmic variability, supporting the interpretation of Complexity as a stimulus‐driven perceptual judgment rather than an emotional or identity‐based evaluation [69].

Complexity showed the strongest cross‐cultural effects of any mechanism. Chinese participants rated music, especially K‐pop and blues, as more complex than other countries. This aligns with research showing culturally conditioned expectations for structural density, tonality, and temporal regularity [54], indicating that complexity is perceived, not inherent, and heavily shaped by cultural familiarity.

Taken together, the findings indicate that Complexity reflects a primarily perceptual–cognitive mechanism. It does not reliably predict strong dislike, nor is it closely tied to identity or social meaning. Instead, it captures differences in how listeners process and organize musical information, shaped by cultural listening habits and expectations. Notably, complexity may also function as a form of skill signaling: music can be experienced as subjectively overwhelming while still eliciting respect for its virtuosity. This ambivalence likely dampens aversive reactions, helping to explain why perceived complexity often leads to evaluative distance rather than outright rejection.

### 4.4 Affective and Structural Excess: Sentimentality and Displeasure

One of the clearest distinctions emerging from our analyses concerns the difference between core emotional aversion—captured by the Displeasure factor—and a qualitatively different response reflecting discomfort with emotional expressivity, captured by the Sentimentality factor. Although both relate to affect, they operate in fundamentally distinct psychological spaces.

Displeasure was the strongest predictor of dislike intensity: higher displeasure ratings reliably accompanied stronger genre aversion. This indicates that displeasure reflects a primary affective rejection, consistent with basic sensory, physiological, or emotional unpleasantness [5, 6, 7]. In contrast, Sentimentality showed the opposite pattern: higher scores predicted weaker dislike intensity. Rather than reflecting strong aversion, this factor appears to capture unease in response to affectively charged or understimulating music [9, 10].

Compared to Displeasure, Sentimentality was higher for gospel, opera, and musicals—genres characterized by expressive vocals, emotional narrative, and strong affective signals. By contrast, Displeasure was higher for metal and rap—a cluster defined by intensity, harshness, or negatively valenced affective content. This suggests that Displeasure arises from music that is inherently unpleasant, whereas Sentimentality reflects a mismatch between the music's emotional expressivity and the listener's preferred emotional range. Some listeners may find this form of emotional expressivity intrusive or cringe‐worthy, consistent with psychological accounts of emotional sensitivity in music perception [7, 18].

Ratings of Sentimentality were significantly higher among Chinese participants than among listeners in Australia, Singapore, and the United States. This suggests that sensitivity to sentimental emotional expressivity—and the social norms that govern emotional expression—varies substantially across cultures. In contrast, Displeasure showed no cross‐cultural variation, implying that basic affective aversion to unpleasant or harsh sounds may be a universal human response, less constrained by cultural display rules or socialization [47].

Taken together, this distinction has rarely been addressed in preference research, which typically collapses diverse affective reactions—such as sensory displeasure, emotional mismatch, or expressive discomfort—into a single liking–disliking dimension. This differentiation opens new pathways for theorizing about emotion, culture, and aversion in musical experience.

### 4.5 Social Excess and Exposure: Social Incongruence and Mainstream

Social identity processes emerged as an important—but multifaceted—source of musical aversion. Although both Social Incongruence and Mainstream orientation involve judgments about the social meaning of music, the results across analyses show that they represent two distinct identity‐based mechanisms with different emotional, genre‐specific, and cultural profiles.

The LMMs demonstrated a clear divergence between the two. Social Incongruence predicted stronger dislike intensity, indicating that when music is perceived as incompatible with one's identity or social environment, it evokes genuine aversion. Listeners appear to reject genres that conflict with their values, self‐concept, or peer group, and this mismatch carries emotional weight [7, 24]. In contrast, perceiving a genre as Mainstream showed no association with dislike intensity, suggesting that mainstream judgments are evaluative rather than affectively charged, functioning more as a symbolic boundary practice or taste signaling rather than a strong emotional reaction [23, 26, 28].

Genre patterns were almost opposing each other between these factors. Social Incongruence was rated especially higher for opera and metal, genres that carry clear social, cultural, or ideological connotations. These findings align with research showing that opera is often viewed as highbrow or elitist [32, 35], whereas metal is associated with strong subcultural identities and controversial, moralizing stereotypes [36, 37, 38]. These results suggest that incongruence arises where music represents social groups or values with which listeners do not identify. In contrast, mainstream ratings were higher for seasonal music and K‐pop—genres marked by wide exposure and cultural saturation [40]. Prior work similarly finds that mainstream status can evoke judgments of banality or overexposure without strong affective rejection [34].

Cross‐cultural results again showed that these mechanisms function differently. Chinese participants scored lower on Social Incongruence, consistent with collectivistic tendencies and lower emphasis on individualized distinction [62], whereas Mainstream evaluations varied by genre across countries, consistent with culturally embedded notions of popularity and saturation [39, 41].

Together, identity‐based dislike is not a single mechanism. Instead, it encompasses Social Incongruence—a deeper, affectively potent rejection of music perceived as inconsistent with one's identity or social milieu—and Mainstream aversion—a culturally contingent evaluation of norm conformity that does not necessarily produce strong dislike.

### 4.6 Cross‐Cultural Listener Profiles of Musical Dislikes

The cross‐cultural analyses indicate that mechanisms of musical dislike differ in their sensitivity to cultural context. Some responses—most notably basic displeasure—appear relatively universal, consistent with evidence that certain affective reactions to acoustic cues are widespread across cultures [47, 50].

In contrast, Sentimentality and Complexity showed pronounced cross‐cultural variation. Chinese participants consistently rated their disliked music as more emotionally intense and structurally complex than listeners from other countries. This aligns with research showing that cultural norms surrounding emotional expression [59], tolerance for emotional intensity [53], and expectations for musical structure [55] shape perceptual and emotional processing.

Social Incongruence varied moderately across countries, consistent with theories emphasizing culturally specific constructions of self and group boundaries [33, 62]. Chinese participants scored lower on Social Incongruence (particularly for opera and indie) and Mainstream metrics, consistent with collectivistic tendencies and lower emphasis on individualized distinction [60, 61]. Perceptions of what counts as “mainstream” were also culturally contingent, reflecting cross‐cultural differences in genre hierarchies and popularity norms [28, 51].

At the individual level, LPA identified three listener types: Emotional Dislikers (high Sentimentality), Socially Driven Dislikers (high Social Incongruence), and Balanced Dislikers (moderate scores across mechanisms). These profiles align with established distinctions between affect‐focused, identity‐focused, and mixed evaluative styles in music preference research [4, 65]. Their prevalence varied across countries, with fewer Socially Driven Dislikers among Chinese participants, fully mirroring the reduced demand for personal identity signaling in collectivistic contexts.

### 4.7 The Primacy of Individual Dispositions Over National Background

Individual differences in musical traits played a substantial role in shaping why participants disliked certain musical genres. Higher musical sophistication and openness to experience were associated with reduced aversion to complex or sentimental music, consistent with findings that expertise and openness increase tolerance for novelty, complexity, and emotional depth [70, 72]. In contrast, individuals with higher cognitive engagement showed stronger dislikes for overly sentimental or overly mainstream music, paralleling research showing that analytically oriented listeners prefer structural clarity and resist predictable or formulaic patterns [17].

Extraverted participants reported stronger aversion to complex or sentimental music, consistent with personality‐based differences in arousal sensitivity [66]. Affective engagement style with music increased general displeasure, whereas social engagement style reduced it—mirroring work showing that socially motivated listeners adopt more tolerant or pragmatic approaches to music [1]. Finally, narrative engagement style predicted stronger dislike due to social incongruence, supporting research that individuals attentive to storytelling and symbolic content are more sensitive to ideological and identity cues in music [71, 73].

Crucially, the models showed that individual traits accounted for more variance in dislike reasons (3.3%) than country‐level differences (1.1%). This aligns with broader cross‐cultural psychology findings that person‐level dispositions can outweigh national cultural differences in predicting survey responses and music perception [56, 57]. However, this low variance at the national level must be interpreted with caution, given the cultural similarity between our Western samples (Australia and the United States) and the likely homogenizing effect of shared socioeconomic strata across these contexts. Overall, musical dislike emerges as a layered phenomenon shaped by both stable individual dispositions and cultural context. Musical dislike is therefore not a single evaluative response, but a psychologically grounded and culturally situated pattern of experience.

### 4.8 Limitations

The Chinese sample was relatively small, increasing uncertainty in cultural contrasts. Moreover, the dislike scale was originally developed in a German context and not formally validated across cultures. Our unbalanced group sizes precluded robust structural modeling checks, meaning some items may have been interpreted differently due to cultural nuances. Additionally, this baseline sample imbalance introduces potential statistical artifacts in our country‐level comparisons. Consequently, the pronounced differences found for China must be interpreted with appropriate caution. To accommodate the unbalanced group sizes across target regions, our mixed‐effects frameworks utilize Type III Wald *F*‐tests with Satterthwaite approximations. While this controls for unequal cell sizes and variance heterogeneity, future work could employ formal causal inference techniques (e.g., propensity score matching) to further account for potential structural confounding between regions.

Beyond sample constraints, certain methodological limitations regarding our statistical approach must be acknowledged. Because our analytical framework relies heavily on advanced exploratory methods—specifically the sequential combination of EGA and factor analysis—the findings are inherently bound to the statistical characteristics of our item pool and data structure. Although EGA has been shown to outperform traditional factor retention heuristics [78], such data‐driven approaches carry the risk of capturing sample‐specific dependencies rather than immutable psychological constructs. Furthermore, for the profile analysis, data had to be aggregated by averaging participants’ factor scores across genres. This cluster‐level aggregation necessarily reduces individual response variance and potentially masks highly genre‐specific aversion profiles, resulting in subgroups that reflect generalized cross‐genre tendencies rather than situational reactions.

To fully rule out these methodology‐driven artifacts, future studies should employ confirmatory factor analysis or confirmatory network analysis on independent, balanced samples with culturally adapted validations. Nevertheless, the fact that our extracted dimensions align remarkably well with prior qualitative typologies, combined with the near absence of differences among Australia, Singapore, and the United States, strongly suggests that the scale captures robust, cross‐culturally stable psychological mechanisms rather than statistical anomalies.

### 4.9 Final Conceptual Synthesis: A New Framework for Understanding Musical Dislike

Musical dislikes arise from fundamentally different psychological mechanisms—affective (Displeasure, Sentimentality), identity‐related (Social Incongruence, Mainstream), cognitive (Complexity), and cultural (modulation of all mechanisms)—each of which displays unique behavioral signatures, genre associations, and cross‐cultural patterns. Operating in tandem as a reciprocal feedback system, these mechanisms operate in parallel, interact through shared psychological pathways, and collectively shape how listeners navigate the musical landscape. These mechanisms produce systematic patterns across behaviors, genres, listener traits, and cultural contexts, revealing that dislike is not the inverse of preference but a structured and meaningful component of musical experience. By showing that aversive responses are multidimensional, interrelated, and tied to both personal dispositions and cultural environments, the study demonstrates that what people reject in music provides crucial insight into how they regulate emotion, negotiate identity, and position themselves within social and cultural worlds. Understanding musical dislikes therefore offers a powerful and complementary lens on the architecture of musical taste, highlighting that to grasp how individuals use music for social distinction and affect regulation, we must also understand what they avoid and why.

## Author Contributions

J.M. and A.E.K. designed the research. K.F. and A.E.K. collected the data. J.M. analyzed the data. J.M. wrote the manuscript. A.E.K. and K.F. revised the manuscript.

## Conflicts of Interest

The authors declare no conflicts of interest.

## Supporting information




**Supporting File 1**: nyas70408‐sup‐0001‐SuppMat.docx

**Supporting File 2**: nyas70408‐sup‐0002‐SuppMat.xlsx

**Supporting File 3**: nyas70408‐sup‐0003‐SuppMat.csv

## Data Availability

The data that support the findings of this study are openly available as Supporting Information.
