# Supplementary material for: Structure of Musical Dislikes: Findings From Multiple Genres and Countries
Source: Ann N Y Acad Sci. 2026 Oct 1;1564(1):e70408. doi: 10.1111/nyas.70408 (PMC13629593; doi:10.1111/nyas.70408)
Supplement: Supplementary file 1 [file NYAS-1564-0-s002.docx]

**Supplement**

**Structure of Musical Dislikes: Findings from Multiple Genres and Countries**

**Participant characteristics**
Participants reported their country of residence, current life circumstances, and financial situation. The largest proportions of participants resided in the United States (n = 458), Australia (n = 253), and Singapore (n = 240), followed by China (n = 55), the United Kingdom (n = 8), Malaysia (n = 7), and other countries (n = 57).

Self-reported life circumstances were measured on a seven-point scale ranging from “among the worst” to “among the best.” Most participants rated their circumstances as average (n = 320) or better than average (n = 415), with additional participants reporting much better than average (n = 207) or among the best (n = 39). Fewer participants indicated worse than average (n = 70), much worse than average (n = 14), or among the worst (n = 13).

Financial situation was assessed using a four-point categorical measure reflecting perceived ability to meet a financial threshold (approximately USD 2,000). Responses were distributed across “certainly not” (n = 152), “probably not” (n = 267), “probably yes” (n = 385), and “certainly yes” (n = 274).

Table 1. Genres and counts per country.

| **Genre** | **AU** | **SG** | **US** | **CN** | **MY** | **UK** | **OTHER** |
| --- | --- | --- | --- | --- | --- | --- | --- |
| Alternative | 6 | 6 | 11 | 1 | 0 | 0 | 4 |
| Aussie Country | 23 | 4 | 6 | 3 | 0 | 0 | 0 |
| Aussie Hip-hop (incl. Drill) | 18 | 4 | 5 | 1 | 0 | 0 | 1 |
| Bluegrass | 5 | 2 | 25 | 2 | 0 | 0 | 1 |
| Blues | 11 | 5 | 20 | 5 | 0 | 0 | 3 |
| Bush ballads/ Bush songs | 23 | 4 | 3 | 3 | 0 | 0 | 0 |
| C-pop (Chinese pop/ Canto-pop/ Mandopop/Mandapop/ Hokkien/ T-pop) | 4 | 14 | 7 | 1 | 0 | 0 | 0 |
| Classical | 31 | 12 | 25 | 1 | 1 | 1 | 0 |
| Country | 39 | 14 | 94 | 1 | 0 | 2 | 7 |
| Dance | 12 | 13 | 22 | 0 | 0 | 0 | 3 |
| Drum and Bass | 11 | 8 | 8 | 3 | 0 | 2 | 3 |
| Easy Listening | 5 | 2 | 11 | 2 | 0 | 1 | 1 |
| Electronic(a)/EDM | 18 | 23 | 50 | 4 | 0 | 0 | 5 |
| Experimental | 2 | 5 | 6 | 5 | 0 | 3 | 2 |
| Folk | 11 | 6 | 26 | 3 | 0 | 0 | 3 |
| Funk | 10 | 5 | 11 | 1 | 1 | 0 | 4 |
| Getai | 1 | 23 | 1 | 1 | 0 | 0 | 0 |
| Gospel | 22 | 12 | 45 | 2 | 0 | 1 | 3 |
| Heavy Metal | 43 | 48 | 63 | 7 | 1 | 0 | 9 |
| Hip-hop | 4 | 11 | 15 | 4 | 1 | 0 | 3 |
| Holiday/Seasonal | 25 | 21 | 40 | 2 | 0 | 0 | 4 |
| House | 5 | 6 | 20 | 0 | 0 | 0 | 4 |
| Indian Pop | 7 | 7 | 8 | 2 | 2 | 0 | 0 |
| Indie | 4 | 2 | 12 | 3 | 0 | 0 | 0 |
| Indie Rock | 7 | 4 | 14 | 2 | 0 | 0 | 3 |
| International/World | 11 | 4 | 8 | 1 | 0 | 0 | 3 |
| J-Pop | 8 | 3 | 13 | 1 | 0 | 1 | 2 |
| Jazz | 18 | 10 | 13 | 2 | 0 | 0 | 2 |
| K-Pop | 25 | 21 | 50 | 4 | 0 | 1 | 7 |
| Latin | 7 | 5 | 14 | 1 | 1 | 1 | 2 |
| Malay pop | 4 | 6 | 4 | 2 | 1 | 0 | 1 |
| Manyao (Mandarin EDM) | 3 | 21 | 2 | 5 | 3 | 0 | 2 |
| Musicals | 19 | 16 | 44 | 4 | 0 | 0 | 3 |
| New Age | 11 | 9 | 11 | 4 | 0 | 0 | 2 |
| Oldies | 4 | 5 | 12 | 1 | 0 | 1 | 0 |
| Opera | 39 | 22 | 63 | 4 | 0 | 3 | 9 |
| Pop | 5 | 4 | 26 | 2 | 0 | 1 | 0 |
| Punk | 18 | 18 | 30 | 5 | 0 | 1 | 3 |
| R&B | 5 | 4 | 11 | 0 | 0 | 0 | 0 |
| Rap | 17 | 20 | 47 | 5 | 2 | 3 | 8 |
| Reggae | 7 | 9 | 12 | 6 | 1 | 1 | 5 |
| Religious (incl. Christian) | 38 | 15 | 72 | 3 | 0 | 0 | 4 |
| Rock | 16 | 20 | 23 | 4 | 0 | 0 | 5 |
| Soul | 8 | 3 | 14 | 2 | 0 | 1 | 1 |
| Soundtracks/ Theme Songs | 7 | 6 | 11 | 3 | 0 | 0 | 2 |
| Techno | 27 | 23 | 49 | 4 | 0 | 0 | 8 |
| Thai EDM | 1 | 4 | 1 | 2 | 1 | 0 | 3 |
| Trap | 14 | 11 | 30 | 2 | 0 | 1 | 2 |
| Xinyao (Xīnjiāpō Gēyáo) | 2 | 8 | 0 | 1 | 0 | 0 | 0 |

Table 2. Items of reasons for musical dislikes. Presented in this order.

| **No.** | **Item** | **Abbreviation** |
| --- | --- | --- |
| 32 | it's not authentic enough | social.not_authentic |
| 12 | it contains too many off-key sounds | music.too_disharmonic |
| 8 | it doesn’t have enough emotional expression | emo.expressionless |
| 29 | it always sounds the same | music.too_uniform |
| 6 | it has no effect on my mood | emo.no_impact |
| 19 | it's not melodic enough | music.too_little_melodious |
| 2 | I can’t move to it | body.missing_danceability |
| 34 | I can't identify with it | social.no_identification |
| 40 | the lyrics are too close to reality | lyrics.too_realistic |
| 23 | it's not rhythmic enough | music.too_unrhythmic |
| 26 | it's too fast | music.too_fast |
| 1 | it’s physically uncomfortable | body.displeasure |
| 4 | it doesn’t touch me | emo.no_feelings |
| 5 | it puts me in a bad mood | emo.bad_mood |
| 24 | it's too structured | music.too_schematic |
| 35 | I associate it with negative experiences | social.bad_experiences |
| 18 | it’s too melodic | music.too_melodious |
| 42 | the emotional content is fake or exaggerated | emo.fake |
| 25 | it's too chaotic | music.too_chaotic |
| 28 | it's too variable | music.too_variable |
| 13 | it uses instruments whose sound I don’t like | music.disliked_instruments |
| 39 | the lyrics are too simple | lyrics.too_simple |
| 36 | my friends dislike it also | social.not_peer_approved |
| 38 | the lyrics are too complex | lyrics.too_complex |
| 17 | it’s too quiet | music.too_soft |
| 22 | it’s too rhythmic | music.too_rhythmic |
| 11 | it doesn’t have enough musical tension | music.too_little_tension |
| 37 | I don't have anything in common with the people who listen to it | social.reject_fanbase |
| 27 | it's too slow | music.too_slow |
| 16 | it’s too loud | music.too_loud |
| 30 | I don't like the way the voice is used | music.bad_vocals |
| 21 | it’s too niche | music.too_niche |
| 33 | I've heard it too often | social.too_often_heard |
| 20 | it’s too close to mainstream | music.too_mainstream |
| 41 | the lyrics are too far from reality | lyrics.too_unrealistic |
| 9 | it's too complex | music.too_complex |
| 31 | I can't relate to the ideology of it | social.incongruent_ideology |
| 14 | it has changed too much | music.too_much_change |
| 3 | it triggers unpleasant feelings in me | emo.bad_feelings |
| 7 | it’s too emotional | emo.too_emotional |
| 15 | it shows too little development | music.too_little_change |
| 10 | it's too simple | music.too_simple |

Table 3. Bridge strength per item.

| **Item** | **Bridge Strength** |
| --- | --- |
| music.too_slow | 0.75121690 |
| body.displeasure | 0.66889701 |
| emo.no_feelings | 0.64549802 |
| social.incongruent_ideology | 0.64482665 |
| music.too_unrhythmic | 0.64230259 |
| music.too_complex | 0.63428965 |
| emo.fake | 0.62877426 |
| music.too_uniform | 0.62273501 |
| emo.bad_feelings | 0.57927248 |
| music.bad_vocals | 0.55817888 |
| music.too_fast | 0.55633895 |
| music.too_little_melodious | 0.54850501 |
| lyrics.too_unrealistic | 0.52744528 |
| lyrics.too_complex | 0.50334745 |
| music.too_little_tension | 0.50168898 |
| lyrics.too_simple | 0.49648619 |
| music.too_schematic | 0.49345467 |
| music.too_niche | 0.47846159 |
| social.not_peer_approved | 0.46637535 |
| emo.expressionless | 0.45350323 |
| music.too_mainstream | 0.45327786 |
| music.too_soft | 0.44262216 |
| music.too_little_change | 0.43889602 |
| music.too_much_change | 0.43773475 |
| music.too_loud | 0.43258116 |
| social.not_authentic | 0.41507083 |
| emo.too_emotional | 0.38959822 |
| social.bad_experiences | 0.36250215 |
| music.too_simple | 0.36192439 |
| music.too_rhythmic | 0.35535873 |
| music.disliked_instruments | 0.35512404 |
| body.missing_danceability | 0.35057946 |
| lyrics.too_realistic | 0.34311964 |
| emo.bad_mood | 0.33412117 |
| emo.no_impact | 0.32801164 |
| music.too_variable | 0.32156382 |
| music.too_chaotic | 0.25971844 |
| music.too_melodious | 0.23024975 |
| social.no_identification | 0.22790860 |
| social.reject_fanbase | 0.18810424 |
| social.too_often_heard | 0.11307696 |
| music.too_disharmonic | 0.09396645 |

Figure S1. Standardized fixed-effect estimates from a linear mixed-effects model predicting reasons (factor scores) with genres. Points represent model estimates; lines indicate 95% confidence.
